# Does transanal drainage tubes placement have an impact on the incidence of anastomotic leakage after rectal cancer surgery? a systematic review and meta-analysis

**DOI:** 10.1186/s12885-024-11990-8

**Published:** 2024-02-24

**Authors:** Yating Liu, Xuhua Hu, Yu Huang, Xu Yin, Pengfei Zhang, Yaoguang Hao, Hongyan Li, Guiying Wang

**Affiliations:** 1 Department of Gastrointestinal Surgery, the Third Hospital of Hebei Medical University, Shijiazhuang, P.R. China; 2 The Second General Surgery, the Fourth Hospital of Hebei Medical University, Shijiazhuang, P.R. China; 3 Department of Gastrointestinal Surgery, the Second Hospital of Hebei Medical University, Shijiazhuang, P.R. China

**Keywords:** Transanal drainage tubes, Rectal cancer, Anastomotic leakage, Prospective studies, Meta-analysis

## Abstract

**Background:**

Whether Transanal drainage tubes (TDTs) placement reduces the occurrence of anastomotic leakage (AL) after rectal cancer (RC) surgery remains controversial. Most existing meta-analyses rely on retrospective studies, while the prospective studies present an inadequate level of evidence.

**Methods:**

A systematic review and meta-analysis of prospective studies on TDTs placement in RC patients after surgery was conducted. The main analysis index was the incidence of AL, Grade B AL, and Grade C AL, while secondary analysis index was the incidence of anastomotic bleeding, incision infection, and anastomotic stenosis. A comprehensive literature search was performed utilizing the databases Cochrane Library, Embase, PubMed, and Web of Science. We recorded Risk ratios (RRs) and 95% confidence intervals (CI) for each included study, and a fixed-effect model or random-effect model was used to investigate the correlation between TDTs placement and four outcomes after RC surgery.

**Results:**

Seven studies (1774 participants, TDT 890 vs non-TDT 884) were considered eligible for quantitative synthesis and meta-analysis. The meta-analysis revealed that the incidence of AL was 9.3% (83/890) in the TDT group and 10.2% (90/884) in the non-TDT group. These disparities were found to lack statistical significance (*P* = 0.58). A comprehensive meta-analysis, comprising four studies involving a cumulative sample size of 1259 participants, revealed no discernible disparity in the occurrence of Grade B AL or Grade C AL between the TDT group and the non-TDT group (Grade B AL: TDT 34/631 vs non-TDT 26/628, *P* = 0.30; Grade C AL: TDT 11/631 vs non-TDT 27/628, *P* = 0.30). Similarly, the incidences of anastomotic bleeding (4 studies, 876 participants), incision infection (3studies, 713 participants), and anastomotic stenosis (2studies, 561 participants) were 5.5% (24/440), 8.1% (29/360), and 2.9% (8/280), respectively, in the TDT group, and 3.0% (13/436), 6.5% (23/353), and 3.9% (11/281), respectively, in the non-TDT group. These differences were also determined to lack statistical significance (*P* = 0.08, *P* = 0.43, *P* = 0.48, respectively).

**Conclusion:**

The placement of TDTs does not significantly affect the occurrence of AL, Grade B AL, and Grade C AL following surgery for rectal cancer. Additionally, TDTs placement does not be associated with increased complications such as anastomotic bleeding, incision infection, or anastomotic stenosis.

**Trial registration:**

PROSPERO: CRD42023427914

## Introduction

Colorectal cancer (CRC) is the most common malignancy of the digestive tract, with the third highest incidence of all malignancies worldwide and the second leading cause of cancer death [1].The incidence of rectal cancer (RC) in China has been on the rise [2]. As part of a comprehensive treatment, RC is currently treated with surgery. Due to the rapid development of Total Neoadjuvant Therapy (TNT), the multimodal approach in the treatment of rectal cancer and various anastomosis and reconstruction techniques in recent years, the treatment of RC with colorectal surgery has made great progress and the incidence of postoperative complications and dysfunction was also significantly reduced in some patients [3]. Nevertheless, anastomotic leakage (AL), a serious complication, remained prevalent [4, 5].

Patients with AL have a poor prognosis, lengthy treatment times, and immense economic and psychological burdens, as well as complications such as peritonitis and sepsis [6].The occurrence of AL will also contribute to a higher local recurrence rate and a lower survival rate [7]. The factors influencing AL are not entirely clear at present. Several studies have shown that TDTs placement after the surgery of RC can reduce the risk of AL [6, 8, 9] or Grade C AL [10], but some studies have shown different results. The placement of TDTs can replace the effect of enterostomy in protecting the anastomosis and avoiding the reoperation, according to some researchers [11]. However, some researchers believe that TDTs placement will not reduce the occurrence of AL but may also cause anastomotic bleeding and intestinal perforation due to mechanical stimulation [6]. Therefore, through a systematic review and meta-analysis of TDTs placement and AL after RC surgery in prospective studies, this study further clarified the internal relationship between them, aiming to provide theoretical references for clinical practice.

## Materials and methods

### Literature search

A comprehensive search was conducted across four prominent literature databases (Web of Science, Embase, Cochrane Library, PubMed) to identify records published in the English language prior to August 15, 2023. We used the terms "Rectal Neoplasms", "Anastomotic Leak", "Prospective Studies" as subject terms. As free words, I used "Rectum tumor", "transanal tube", "Transanal drainage tube", "Anastomotic Leakage", "Leaks, Anastomotic", "Prospective Study", "Study, Prospective" etc. In order to enhance the efficacy of retrieval outcomes, we integrated the subject term with an unrestricted term. Due to the absence of the subject term "Transanal drainage tube" in PubMed, an unrestricted term search was conducted for the object. To prevent research from being missed, further relevant studies were identified by manually searching references in the online databases and systematic reviews that have been previously published. The literature retrieval processes were comprehensively outlined in Table 1.

**Table 1:** Literature search strategy

Database	Search build	Occurrences
PUBMED	("transanal tube"[Title/Abstract] OR "anal tube"[Title/Abstract] OR "catheter drainage"[Title/Abstract] OR "transanal drainage tube"[Title/Abstract] OR "transanal tube"[Title/Abstract] OR "transanal decompression tube"[Title/Abstract] OR "transanal drainage tubes"[Title/Abstract] OR "transanal drainage"[Title/Abstract] OR "rectal tube drainage"[Title/Abstract] OR "transanal indwelling"[Title/Abstract] OR "transanal stent"[Title/Abstract] OR "rectal tube"[Title/Abstract]) AND ("Rectal Neoplasms"[MeSH Terms] OR ("Rectal Neoplasms"[Title/Abstract] OR "rectum tumor"[Title/Abstract] OR "neoplasm rectal"[Title/Abstract] OR "rectal neoplasm"[Title/Abstract] OR "rectum neoplasms"[Title/Abstract] OR "neoplasm rectum"[Title/Abstract] OR "rectum neoplasm"[Title/Abstract] OR "rectal tumors"[Title/Abstract] OR "rectal tumor"[Title/Abstract] OR "tumor rectal"[Title/Abstract] OR "neoplasms rectal"[Title/Abstract] OR "cancer of rectum"[Title/Abstract] OR "rectum cancers"[Title/Abstract] OR "rectal cancer"[Title/Abstract] OR "cancer rectal"[Title/Abstract] OR "rectal cancers"[Title/Abstract] OR "rectum cancer"[Title/Abstract] OR "cancer rectum"[Title/Abstract] OR "cancer of the rectum"[Title/Abstract])) AND ("Anastomotic Leak"[MeSH Terms] OR ("Anastomotic Leak"[Title/Abstract] OR "anastomosis leakage"[Title/Abstract] OR "anastomotic leaks"[Title/Abstract] OR "leak anastomotic"[Title/Abstract] OR "leaks anastomotic"[Title/Abstract] OR "anastomotic leakage"[Title/Abstract] OR "anastomotic leakages"[Title/Abstract] OR "leakage anastomotic"[Title/Abstract] OR "leakages anastomotic"[Title/Abstract])) AND ("Prospective Studies"[MeSH Terms] OR "Prospective Studies"[Title/Abstract] OR "prospective study"[Title/Abstract] OR "studies prospective"[Title/Abstract] OR "study prospective"[Title/Abstract])	10
EMBASE	('rectum tumor'/exp OR 'Rectum tumor'/:ab,ti OR 'Rectal Neoplasms'/:ab,ti OR 'Neoplasm, Rectal'/:ab,ti OR 'Rectal Neoplasm'/:ab,ti OR 'Rectum Neoplasms'/:ab,ti OR 'Neoplasm, Rectum'/:ab,ti OR 'Rectum Neoplasm'/:ab,ti OR 'Rectal Tumors'/:ab,ti OR 'Rectal Tumor'/:ab,ti OR 'Tumor, Rectal'/:ab,ti OR 'Neoplasms, Rectal'/:ab,ti OR 'Cancer of Rectum'/:ab,ti OR 'Rectum Cancers'/:ab,ti OR 'Rectal Cancer'/:ab,ti OR 'Cancer, Rectal'/:ab,ti OR 'Rectal Cancers'/:ab,ti OR 'Rectum Cancer'/:ab,ti OR 'Cancer, Rectum'/:ab,ti OR 'Cancer of the Rectum'/:ab,ti) AND ('transanal tube'/:ab,ti OR 'anal tube'/:ab,ti OR 'catheter drainage'/:ab,ti OR 'Transanal drainage tube'/:ab,ti OR 'transanal tube'/:ab,ti OR 'Transanal decompression tube'/:ab,ti OR 'Transanal drainage tubes'/:ab,ti OR 'Transanal drainage'/:ab,ti OR 'rectal tube drainage'/:ab,ti OR 'Transanal indwelling'/:ab,ti OR 'Transanal stent'/:ab,ti OR 'Rectal tube'/:ab,ti) AND ('Anastomosis Leakage'/exp OR 'Anastomosis Leakage'/:ab,ti OR 'Anastomotic Leak'/:ab,ti OR 'Anastomotic Leaks'/:ab,ti OR 'Leak, Anastomotic'/:ab,ti OR 'Leaks, Anastomotic'/:ab,ti OR 'Anastomotic Leakage'/:ab,ti OR 'Anastomotic Leakages'/:ab,ti OR 'Leakage, Anastomotic'/:ab,ti OR 'Leakages, Anastomotic'/:ab,ti) AND ('Prospective Study'/exp OR 'Prospective Study'/:ab,ti OR 'Prospective Studies'/:ab,ti OR 'Studies, Prospective'/:ab,ti OR 'Study, Prospective'/:ab,ti)	65
COCHRANE LIBRARY	("Rectal Neoplasms"[Mesh descriptor] OR "Rectal Neoplasms":ti,ab,kw OR "Rectum tumor":ti,ab,kw OR "Neoplasm, Rectal":ti,ab,kw OR "Rectal Neoplasm":ti,ab,kw OR "Rectum Neoplasms":ti,ab,kw OR "Neoplasm, Rectum":ti,ab,kw OR "Rectum Neoplasm":ti,ab,kw OR "Rectal Tumors":ti,ab,kw OR "Rectal Tumor":ti,ab,kw OR "Tumor, Rectal":ti,ab,kw OR "Neoplasms, Rectal":ti,ab,kw OR "Cancer of Rectum":ti,ab,kw OR "Rectum Cancers":ti,ab,kw OR "Rectal Cancer":ti,ab,kw OR "Cancer, Rectal":ti,ab,kw OR "Rectal Cancers":ti,ab,kw OR "Rectum Cancer":ti,ab,kw OR "Cancer, Rectum":ti,ab,kw OR "Cancer of the Rectum":ti,ab,kw) AND ("transanal tube":ti,ab,kw OR "anal tube":ti,ab,kw OR "catheter drainage":ti,ab,kw OR "Transanal drainage tube":ti,ab,kw OR "transanal tube":ti,ab,kw OR "Transanal decompression tube":ti,ab,kw OR "Transanal drainage tubes":ti,ab,kw OR "Transanal drainage":ti,ab,kw OR "rectal tube drainage":ti,ab,kw OR "Transanal indwelling":ti,ab,kw OR "Transanal stent":ti,ab,kw OR "Rectal tube":ti,ab,kw) AND ("Anastomotic Leak"[Mesh descriptor] OR "Anastomotic Leak":ti,ab,kw OR "Anastomosis Leakage":ti,ab,kw OR "Anastomotic Leaks":ti,ab,kw OR "Leak, Anastomotic":ti,ab,kw OR "Leaks, Anastomotic":ti,ab,kw OR "Anastomotic Leakage":ti,ab,kw OR "Anastomotic Leakages":ti,ab,kw OR "Leakage, Anastomotic":ti,ab,kw OR "Leakages, Anastomotic":ti,ab,kw) AND ("Prospective Study"[Mesh descriptor] OR "Prospective Studies":ti,ab,kw OR "Prospective Study":ti,ab,kw OR "Studies, Prospective":ti,ab,kw OR "Study, Prospective":ti,ab,kw)	21
WEB OF SCIENCE	("Rectal Neoplasms":TS OR "Rectum tumor":TS OR "Neoplasm, Rectal":TS OR "Rectal Neoplasm":TS OR "Rectum Neoplasms":TS OR "Neoplasm, Rectum":TS OR "Rectum Neoplasm":TS OR "Rectal Tumors":TS OR "Rectal Tumor":TS OR "Tumor, Rectal":TS OR "Neoplasms, Rectal":TS OR "Cancer of Rectum":TS OR "Rectum Cancers":TS OR "Rectal Cancer":TS OR "Cancer, Rectal":TS OR "Rectal Cancers":TS OR "Rectum Cancer":TS OR "Cancer, Rectum":TS OR "Cancer of the Rectum":TS) AND ("transanal tube":TS OR "anal tube":TS OR "catheter drainage":TS OR "Transanal drainage tube":TS OR "transanal tube":TS OR "Transanal decompression tube":TS OR "Transanal drainage tubes":TS OR "Transanal drainage":TS OR "rectal tube drainage":TS OR "Transanal indwelling":TS OR "Transanal stent":TS OR "Rectal tube":TS) AND ("Anastomotic Leak":TS OR "Anastomosis Leakage":TS OR "Anastomotic Leaks":TS OR "Leak, Anastomotic":TS OR "Leaks, Anastomotic":TS OR "Anastomotic Leakage":TS OR "Anastomotic Leakages":TS OR "Leakage, Anastomotic":TS OR "Leakages, Anastomotic":TS) AND ("Prospective Studies":TS OR "Prospective Study":TS OR "Studies, Prospective":TS OR "Study, Prospective":TS)	28

### Outcomes of interest and definition

Anastomotic leakage was defined as a defect in the intestinal wall at the anastomotic site that allows communication between the intraluminal and extraluminal compartments [12]. Grade A AL: Patients are usually free of clinical symptoms and laboratory abnormalities. There is no necessity for therapeutic intervention, as the patient exhibits clinical wellness. Grade B AL: Patients usually have abdominal pain, abdominal distension, and fever, and intra-operatively placed pelvic drains may discharge turbid/purulent or fecal fluid. The patient often needs aggressive interventions such as the implementation of antibiotic therapy, along with the utilization of pelvic drain placement or transanal lavage. Grade C AL: Patients are often quite ill and require operative re-laparotomy [12]. Anastomotic bleeding was defined as a notable decrease in hemoglobin and active and the presence of ongoing rectal bleeding were not associated with any other cause [13]. Anastomotic stenosis was defined as the 12-diameter mm colonoscopy cannot passes through the benign narrowing of the anastomosis. Incision infection was defined as an inflammation in the incision and bacterial growth in the incision secretion culture.

### Study selection

We used inclusion criteria and exclusion criteria to screen literature related to this study. The following criteria were used to select the studies for the meta-analysis: (1) published as an original article; (2) belonged to prospective study; (3) evaluated the association between the placement of TDTs and the occurrence of AL after RC surgery; (4) given the number of participants.; (5) the risk estimates are presented alongside their respective 95% confidence intervals (95% CI). In the present study, we will proceed to exclude research investigations that are relevant to any of the following categories: (1) Emergency surgery; (2) review paper; (3) animal trials; (4) conference papers; (5) the full text is not accessible; (6) the data cannot be extracted.

### Data extraction and quality assessment

The entirety of the articles' content was thoroughly examined during the review process. Data extraction and full-text review were carried out independently based on the preferred reporting items for systematic reviews, A Measurement Tool to Assess Systematic Review 2 (AMSTAR2) and meta-analyses (PRISMA) guidelines by two reviewers and the inconsistencies were rectified by a third author. To eliminate any instances of duplication, the extracted study will be imported into the Endnote Software X9.0, after which the titles and abstracts will be reviewed by two researchers. Furthermore, adherence to the MOOSE (meta-analysis of observational studies in epidemiology) guidelines is recommended [14]. The collection of data was carried out utilizing standardized forms that were developed by the research team. The information included in the data extraction will be as follows: year of publication, design of study, authors, the quantity of individuals participating in the study. Furthermore, we also conducted an examination of the clinical data and indicators: (1) design of study (Randomized controlled trials vs. Prospective Cohort study vs. Non-randomized controlled trials); (2) case/participants; (3) area (Asia vs. Europe); (4) publication year (≤ 2015 vs. > 2015); (5) quality score (≤ 7 vs. > 7).

### Statistical analysis

The meta-analysis was conducted using the Review Manager 5.3 and Stata15.0 software programs. Given that this study obtained binary data from prospective studies, the effect size was determined by employing the risk ratio (RR) calculation. The I^2^ index and Cochran's Q tests were utilized to quantify the levels of incoherence and heterogeneity among the studies, respectively. The I^2^ index was assessed as a metric for evaluating the extent of heterogeneity across the studies. The data was examined through the utilization of a fixed-effect model in instances where there was an absence of heterogeneity (*P* value from the $\chi$ ^2^ test > 0.05 and I^2^ statistic value < 50%) among studies, while a random-effects model was employed when heterogeneity was present (*P* value from the $\chi$ ^2^ test ≤ 0.05 and I^2^ statistic value ≥ 50%) among studies. To explore potential causes of heterogeneity, sensitivity and subgroup analyses were conducted. Multiple confounding factors were present, including design of the study, quality score, area, and publication year. In addition, sensitivity analyses were carried out to evaluate the robustness of the primary results. Moreover, Egger's correlation tests accounted for the influence of publication bias, and a *P* value < 0.05 (*) was deemed to be statistically significant [15].

## Results

### Search results

**Fig. 1:**
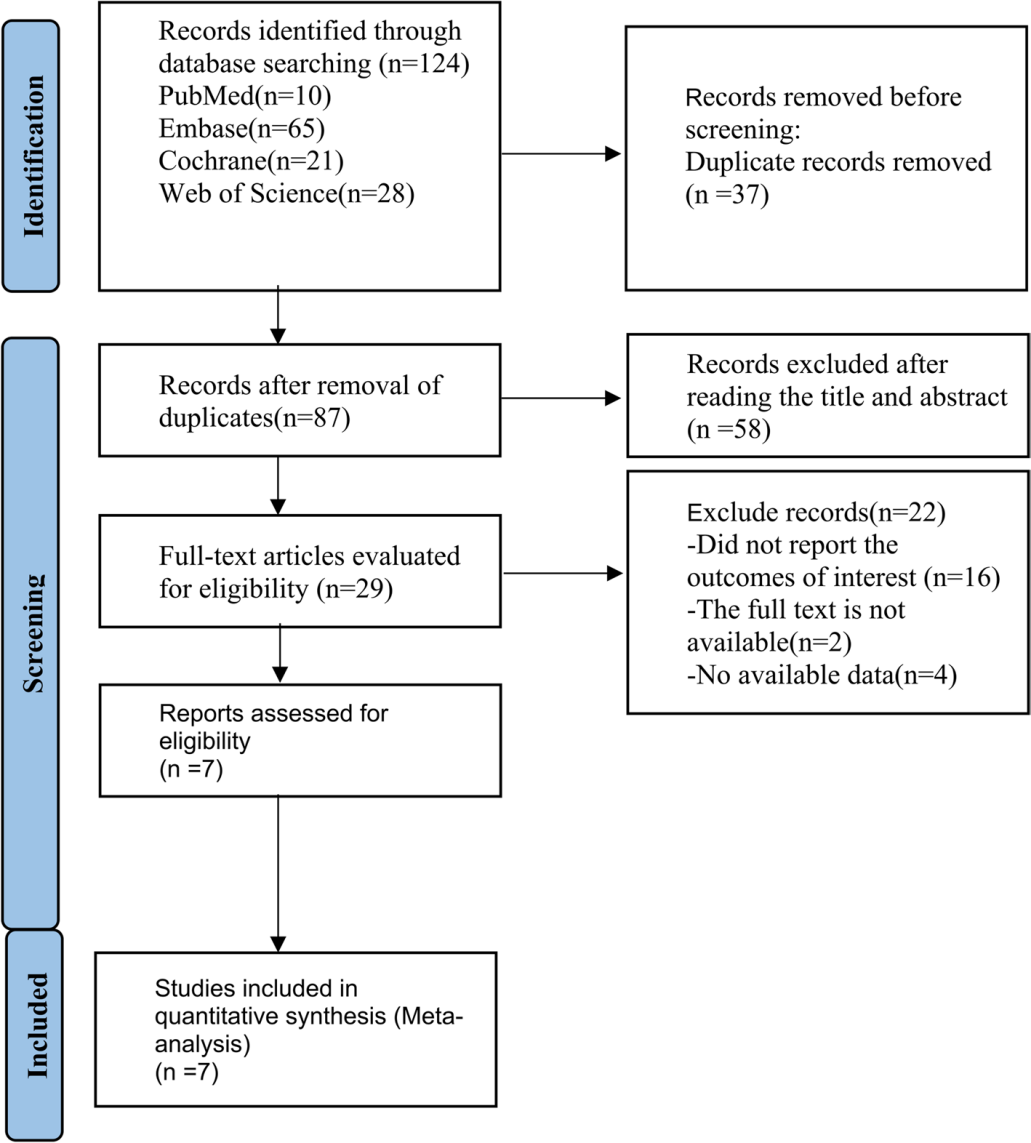
Description of the entire process from literature retrieval to the selection of 7 target articles

From the initial literature, 124 relevant studies were identified (10 studies from PubMed, 21 studies from Cochrane Library, 65 studies from Embase, and 28 studies from Web of Science). The first stage involved the elimination of duplicate articles based solely on titles among predefined databases. Due to duplication, 37 articles were excluded, leaving 87 articles for screening based on titles and abstracts. As well as the 58 studies we excluded, we also excluded studies in animals, case reports, and review articles. 29 studies were reviewed comprehensively. 16 articles were excluded for not reporting relevant results, 2 articles were excluded from the analysis due to unavailability of the full text, and 4 articles were excluded since data was not available. Ultimately, we included 7 articles including 1774 participants between 2006 and 2022 in our meta-analysis [4, 9, 13, 16–18]. Fig. 1 illustrates the process of literature retrieval.

### Study characteristics, and quality assessment

Table 2 provided a comprehensive overview of the key attributes of the studies that were incorporated. A total of 1774 participants were involved in the 7 studies published between 2006 and 2022. These studies were carried out 1 in Japan, 1 in Denmark, 1 in France, and 4 in China. In addition, 1 was a non-randomized controlled trial, 4 studies were randomized controlled trials, and 2 were Prospective cohort studies. And TDTs placement has no inherent relationship with the occurrence of AL after RC surgery in all studies. Each study successfully adhered to all criteria pertaining to the avoidance of selection and outcome bias.

**Table 2:** Characteristics of included studies

Author	Year	Nation	Age (year)	Sample size (tube/ no tube)	Study type	NOS	Tumor location (Above the anal verge)	Type of surgery	Stapling technique	The rate of AL		Type of tube	Indwelling time of tube (days)	Position of tube (above the anastomosis)	Decision to remove	Diverting stoma		Preoperative chemotherapy or radiotherapy	
										tube	no tube					TDT	Non-TDT	TDT	Non-TDT
Zhao et al	2013	China	Unmentioned	81/77	nRCT	≤ 7	Unmentioned	LAR	Single- or double-stapler technique	2/81	7/77	A 26Fr rubber drainage tube	5–6 POD	3-5 cm	b	Excluded		Excluded	
Xiao et al	2011	China	46–70	200/198	RCT	> 7	≤ 15 cm	LAR	Double-stapling anastomosis	8/200	19/198	A silicone drainage tube	5–7 POD	Unmentioned	Unmentioned	Excluded		Excluded	
Tamura et al	2021	Japan	39–91	79/78	RCT	> 7	≤ 15 cm	LLAR	Double stapling technique	6/79	8/78	A 20-24Fr Malecot latex tubes	5 POD	3–5 cm	a	34/79	37/78	10 d/79	19 d/78
Challine et al	2020	France	48.2–76.2	72/72	PCS	≤ 7	Unmentioned	LLAR	Double-stapling technique or handsewn	25/72	16/72	A 22Fr Foley catheter	4 POD	Unmentioned	Unmentioned	0/72	72/72	41 d/72	47 d/72
Bu¨low et al	2006	Denmark	37–90	98/96	RCT	> 7	≤ 15 cm	Unmentioned	Unmentioned	14/98	8/96	A transanal silicone stent	Unmentioned	Unmentioned	Unmentioned	44/98	45/96	Excluded	
Zhao et al	2021	China	52–69	280/280	RCT	> 7	5.6-9 cm	LLAR	Double-stapling technique	18/280	19/280	A 28Fr silicone tube	3–7 POD	5 cm	a	72/280	89/280	12c/480	16c/480
Liang et al	2022	China	42.9–70.2	80/83	PCS	≤ 7	0.5–8.8 cm	Unmentioned	Unmentioned	8/80	10/83	A novel endo-luminal balloon-assisted drainage (EBAD)	5–6 POD	5 cm	b	0/80	83/83	43d/80	53 d/83

*RCT* Randomized controlled trials, *nRCT* non-randomized controlled trials, *NOS* The Newcastle–Ottawa Scale, *PCS* Prospective Cohort study, *POD* postoperative day, *LAR* low anterior resection, *LLAR* laparoscopic low anterior resection

a. if the patients had no signs of AL

b. if fecal discharge and gas were continuously observed in transanal drainage fluid but not in the abdominal drainage fluid following the recovery of gastrointestinal function

c. Preoperative chemotherapy

d. Neoadjuvant chemoradiotherapy

### TDT placement and AL after RC surgery risk

**Fig. 2:**
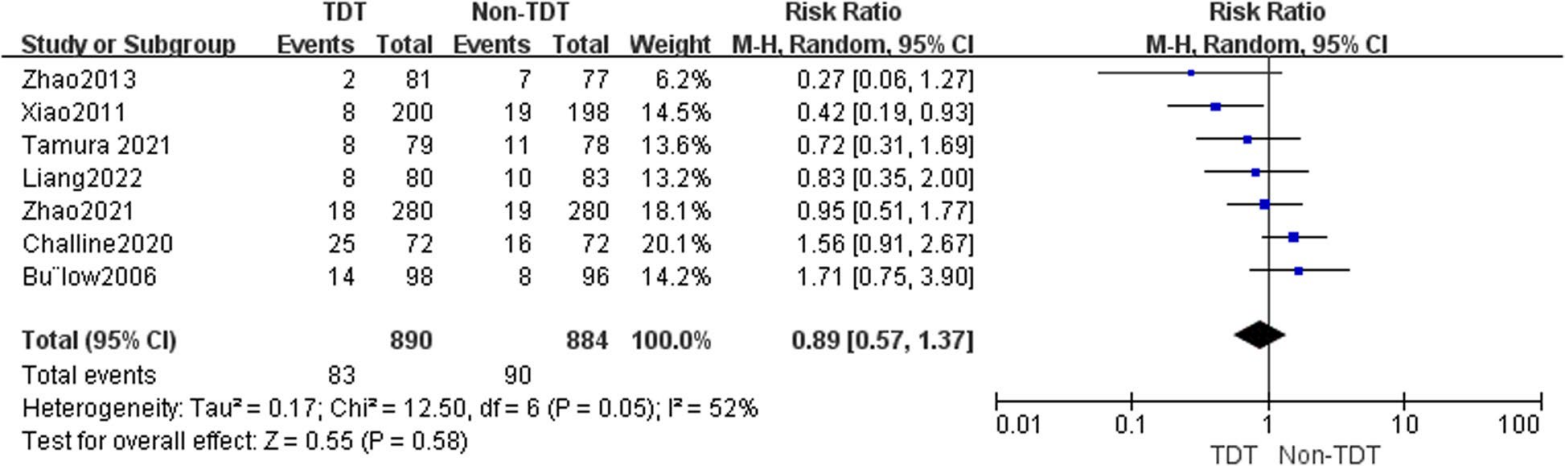
A random-effect model was used to analyze the RRs of 7 articles to compare the association between transanal drainage tubes placement and the occurrence of anastomotic leakage after rectal cancer surgery. RR = 0.89, 95%CI 0.57–1.37, *P* = 0.58

**Fig. 3:**
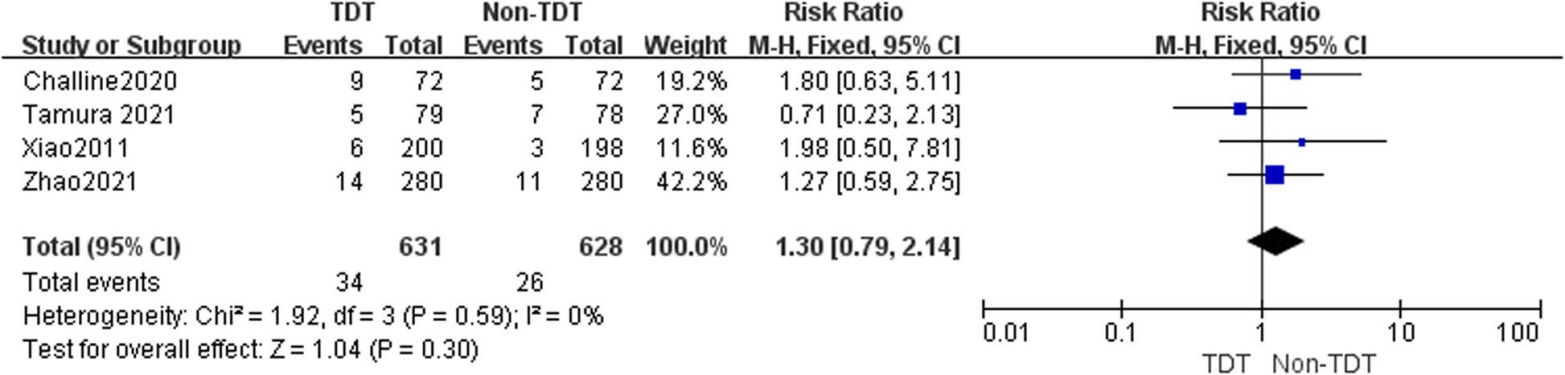
A fixed-effect model was used to analyze the RRs of 4 articles to compare the association between transanal drainage tubes placement and the occurrence of Grade B anastomotic leakage after rectal cancer surgery. RR = 1.30, 95%CI 0.79–2.14, *P* = 0.30

**Fig. 4:**
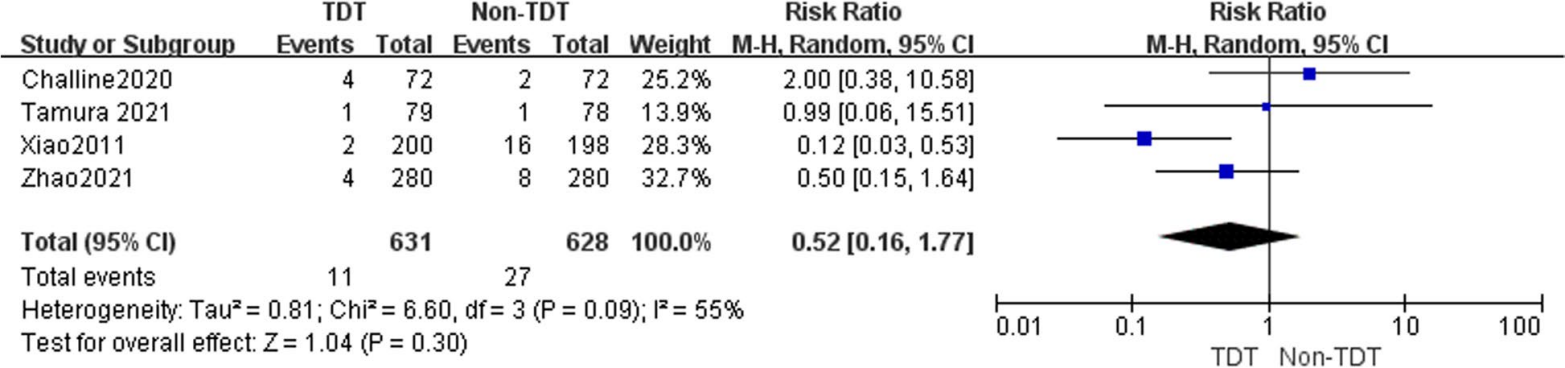
A random-effect model was used to analyze the RRs of 4 articles to compare the association between transanal drainage tubes placement and the occurrence of Grade C anastomotic leakage after rectal cancer surgery. RR = 0.52, 95%CI 0.16–1.77, *P* = 0.30

In Fig. 2, we extracted RRs from 7 studies after multivariable adjustment. We analyzed the data using a random-effects model to compare the association between TDTs placement and the occurrence of AL after RC surgery because of the presence of heterogeneity (*P* = 0.05, $\mathrm{\rm I}$ ^2^ = 52%). The meta-analysis revealed that the occurrence of AL was 9.3% (83/890) in the TDT group and 10.2% (90/884) in the non-TDT group. Upon thorough analysis of the combined results from all tests, it was ascertained that there exists no statistically significant association between the placement of TDTs and the incidence of AL following RC surgery (RR = 0.89, 95%CI 0.57–1.37, *P* = 0.58). Four studies [4, 9, 19] were identified that reported the occurrence of Grade B AL and Grade C AL, which were subsequently subjected to analysis. Upon analysis of the data on Grade B AL, the outcomes of the heterogeneity test indicated no statistically significant level of heterogeneity (*P* = 0.59, $\mathrm{\rm I}$ ^2^ = 0%), thus leading to the adoption of the fixed-effect model. The findings from the meta-analysis indicate that the occurrence of Grade B AL in the TDT group was 5.4% (34/631), which did not exhibit a statistically significant disparity when compared to the 4.1% (26/628) observed in the non-TDT group (RR = 1.30, 95%CI 0.79 -2.14, *P* = 0.30) (Fig. 3). The heterogeneity test indicated statistically significant heterogeneity in the data on Grade C AL (*P* = 0.09, I^2^ = 55%), leading to the adoption of the random-effects model. Similar to the result of Grade B AL, the results of the meta-analysis demonstrate that the prevalence of Grade C AL in the TDT group was 1.7% (11/631), which did not display a statistically significant difference when compared to the (4.3% (27/628) observed in the non-TDT group (RR = 0.52, 95%CI: 0.16 ~ 1.77,* P* = 0.30) (Fig. 4).

### Subgroup

Subgroup analyses were conducted by area, design of the study, publication year, and quality score (Table 3). Initially, a subgroup analysis was performed according to area. The findings from the Asian subgroup (RR = 0.68,95%CI 0.47 ~ 1.00, *P* = 0.05), comprising five studies [4, 9, 13, 16, 18], indicated that the placement of TDTs effectively prevented AL. Conversely, the results from the European subgroup (RR = 1.61,95%CI 1.03 ~ 2.52, *P* = 0.04), consisting of two studies [17, 19], demonstrated a significant correlation between TDTs placement and a heightened occurrence of AL. Moreover, the subgroup analyses concerning variables such as the design of study, publication year, and quality score resulted in inconclusive findings (*P* = 0.17, *P* = 0.38, *P* = 0.92, respectively). Detailed results of the subgroup analysis are presented in Table 3.

**Table 3:** Subgroup analyses of TDTs placement and anastomotic leakage after rectal cancer surgery risk

Group	Studies (*n*)	RR (95% CI)	*P*	Heterogeneity test	
				***P***	***I2* (%)**
Total	7	0.89(0.57–1.37)	0.58	0.05	52
Design					
Randomized controlled trials	4	0.84(0.49–1.45)	0.53	0.11	51
Non-randomized controlled trials	1	0.27(0.06–1.27)	0.10	NA	NA
Prospective Cohort study	2	1.26(0.70–2.27)	0.45	0.23	32
Area					
Asia	5	0.68(0.47–1.00)	0.05	0.39	3
Europe	2	1.61(1.03–2.52)	0.04	0.85	0
Publication year					
≤ 2015	3	0.64(0.21–1.96)	0.43	0.02	74
> 2015	4	1.08(0.75–1.53)	0.69	0.36	7
Quality score					
≤ 7	3	0.89(0.38–2.08)	0.78	0.07	62
> 7	4	0.84(0.49–1.45)	0.53	0.11	51

### TDTs placement and other clinic outcomes risk

In this study, the correlation between the placement of TDTs and anastomotic bleeding, incision infection, and anastomotic stenosis was further investigated. Due to the lack of significant heterogeneity among the studies, a fixed-effect model was used. Studies found no statistically significant association between the placement of TDTs and anastomotic bleeding (RR = 1.77, 95%CI 0.94 -3.33, *P* = 0.08), incision infection (RR = 1.24, 95%CI 0.73 -2.09, *P* = 0.43), or anastomotic stenosis (RR = 0.73, 95%CI 0.30 -1.77, *P* = 0.48). The comprehensive findings are presented in Table 4.

**Table 4:** TDTs placement and other clinic outcomes after rectal cancer surgery risk

Index	Studies	sample size	Heterogeneity test	analytical model	RR	95%CI	*P*
anastomotic bleeding	4	876	*P* = 0.45, I2 = 0%	Fixed Mantel-Haenzel	1.77	0.94 ~ 3.33	0.08
incision infection	3	713	*P* = 0.67, I2 = 0%	Fixed Mantel-Haenzel	1.24	0.73 ~ 2.09	0.43
anastomotic stenosis	2	561	*p* = 0.76, I2 = 0%	Fixed Mantel-Haenzel	0.73	0.30 ~ 1.77	0.48

### Sensitivity analysis

**Fig. 5:**
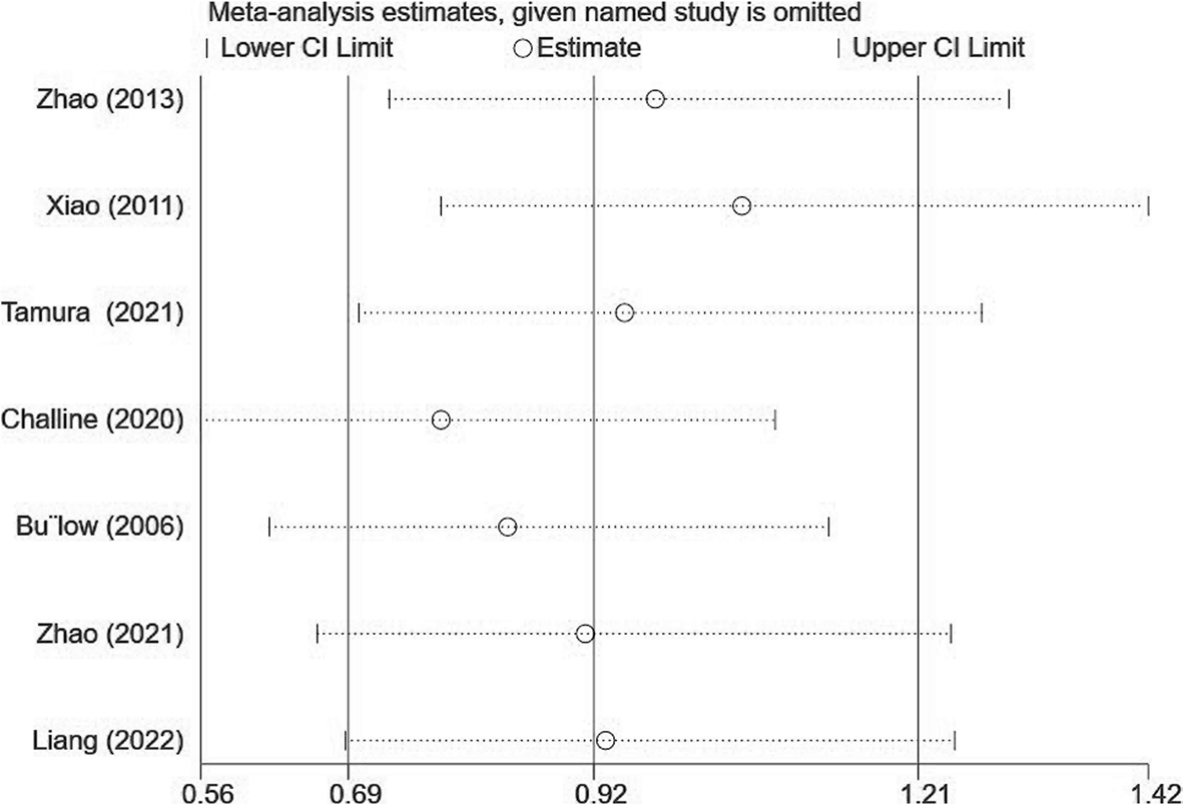
Sensitivity analyses were performed to investigate potential sources of heterogeneity and showed the main result was robustness. The overall results ranged from 0.69(95%CI = 0.56–0.78) to 1.21(95%CI = 1.08–1.42)

To investigate potential sources of heterogeneity, a sensitivity analysis was conducted. Fig. 5 shows the sensitivity analysis results. Except for any individual study, the collective findings exhibited a range of 0.69(95%CI = 0.56–0.78) to 1.21(95%CI = 1.08–1.42). The findings of the study indicate that the exclusion of a single study did not yield any significant disparity between the combined RR and the total RR. This suggests that the placement of TDTs following RC surgery does not exhibit any correlation with a reduced occurrence of AL. As a result, the main result is robustness.

### Publication bias

**Fig. 6:**
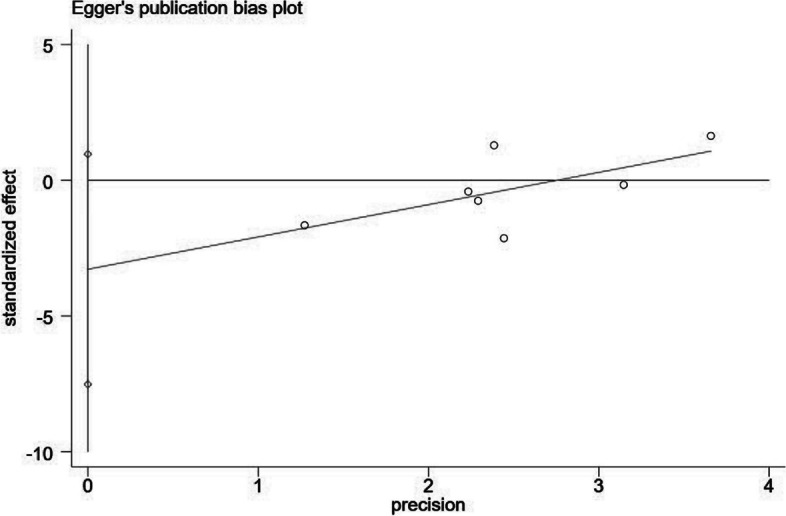
Egger test and Egger test plot were performed to confirm that there was no significant publication bias between the placement of transanal drainage tubes and the occurrence of anastomotic leakage after rectal cancer surgery. *P* = 0.10

To identify the presence of publication bias within the studies that were included, both the Egger test and Egger test plot were employed (Fig. 6). The analysis concluded that there was no substantial evidence of publication bias between the placement of TDTs and the occurrence of AL after RC surgery by Egger's test (*P* = 0.10).

## Disscussion

Currently, a variety of adjuvant therapy techniques and anastomosis methods are used to treat RC, which results in a higher rate of Sphincter Preserve. However, the incidence of AL after RC surgery is still at a high level. Therefore, a clear understanding of the risk factors and protective factors of AL can bring great benefits to patients. The TDTs is used to drain the proximal intestinal contents and reduce the stimulation of the anastomoses. It can reduce intestinal cavity pressure and the tension of anastomoses. However, there is no consensus on whether the placement of TDTs can reduce the occurrence of AL.

AL following RC surgery cannot be reduced with the placement of TDTs, according to 7 prospective studies in this study. At the level of the original study, according to Tumura [16] and Zhao [4] there was no statistical significance between the placement of TDTs and AL, which is consistent with our findings. Meanwhile, the study of Xiao [9] and Zhao [13] demonstrated that TDTs placement was a protective factor for AL. Additionally, meta-analyses of the placement of TDTs and AL after RC surgery have had inconsistent results. A meta-analysis of Deng [20] found that TDTs placement reduced AL incidence in low-risk patients (OR = 0.29, 95%CI = 0.13–0.63, *P* = 0.002), but not in high-risk patients undergoing neoadjuvant treatment. The meta-analysis conducted by Zhao [10]found no significant association between the placement of TDTs and the prevalence of AL. However, it did reveal a reduction in the occurrence of Grade C AL (RR = 0.33, 95%CI = 0.11–1.01, *P* = 0.05). The discrepancies in the results between Deng [20], Zhao [10], and this study may be attributed to the inclusion of different types of studies and Differences in sample size. Deng [20] included both prospective and retrospective studies, this study included prospective studies, and Zhao [10] only included randomized controlled trials. In Guo's [21] subgroup analysis of the meta-analysis, it was determined that TDTs placement did not exhibit a significant association with the low incidence of AL in randomized controlled trials. However, in observational studies, there was a notable association between TDTs placement and the occurrence of low AL. This finding underscores the influence of study design on the obtained results. Among the seven original papers included by Deng [20] it is noteworthy that only three of them were prospective studies. Consequently, the divergent conclusion reached by Deng's [20] study in comparison to the present study can plausibly be attributed to the heterogeneity of results arising from the inclusion of distinct study types. This study exhibits a degree of resemblances to the studies conducted by Deng [20] and Zhao [10]. Nevertheless, Deng's [20] research primarily centers on retrospective studies. The limited evidentiary value of retrospective cohort studies hinders the broad applicability of their findings. Despite the inclusion of the most rigorous randomized controlled trials in Zhao's [10] study, it was relying solely on three primary research papers. In contrast, this study incorporated seven prospective studies. In comparison to Deng's [20] study, the prospective studies integrated into this study entail rigorous data quality control during case screening. This practice serves to mitigate the bias arising from case–control studies to a certain degree, thereby enhancing the reliability of the findings. Furthermore, it encompassed a greater volume of original literature and a larger sample size than Zhao’s [10] study. As a result, this study provides a higher level of evidence and relatively more reliable outcomes.

AL was categorized into one of three grades (Grade A, B, or C) based on its influence on clinical management [12]. Presently, there is a consensus within the academic community regarding the placement of TDTs to alleviate the severity of AL. When AL ensues, the anal sphincter frequently persists in contracting because of inflammation, pain, and other causative factors. Furthermore, AL frequently manifests during the initial postoperative phase, when the intestinal function has not been restored, and the intestinal contents cannot be eliminated in time, resulting in intestinal high pressure. Physical stimulation caused by high pressure in the intestinal cavity and chemical stimulation caused by intestinal contents is not conducive to the healing of the AL. Drainage of intestinal contents by placing TDTs reduces pressure in the lumen and promotes fecal excretion [9, 22], thus promoting recovery of AL. The findings of this meta-analysis indicate that there is no significant correlation between TDTs placement and a reduced occurrence of AL following RC surgery (RR = 0.89, 95%CI 0.57–1.37, *P* = 0.58). Considering the following three factors, the relationship between TDTs placement and the incidence of different grades AL was analyzed: (1) Distinct grades of AL necessitate distinct clinical management principles, (2) Grade C AL is of significant concern, as it necessitates a subsequent surgical intervention and escalates the likelihood of restomy and other postoperative complications, (3) TDTs placement can reduce the severity of AL. Given that only four studies in the original literature included recorded the detailed incidence of AL across all levels, it is noteworthy that two out of these four studies did not document the occurrence of Grade A AL. As a result, the present study directed its analysis towards Grade B AL and Grade C AL, while excluding Grade A AL from consideration. The findings of this research indicate that the implementation of TDTs does not result in a decrease in the occurrence of Grade B AL or Grade C AL. Based on the analysis of data from three randomized controlled trials, Zhao’s study determined that the p-value for the association between TDTs placement and the occurrence of Grade C AL was 0.05. Consequently, the researchers of the study of Zhao [10] reached the determination that the placement of TDTs could potentially yield positive outcomes in mitigating Grade C AL. However, Zhao’s [10]study did not yield any statistically significant association between the placement of TDTs and the mitigation of Grade B AL. This research group holds a dissenting perspective on the notion that the implementation of TDTs is incapable of diminishing the occurrence of minor Grade B AL, yet it can effectively mitigate the prevalence of severe Grade C AL, while nor does Zhao’s [10] article offer an explanation for the possible underlying mechanism. As a result, this study augmented the sample size and arrived at an alternative conclusion, namely, the placement of TDTs does not exhibit no correlation with the low occurrence of Grade C AL. This finding suggests that while the placement of TDTs may mitigate the severity of AL, it does not have a significant impact on the occurrence rate of AL.

Furthermore, the present study revealed that the placement of TDTs did not result in a higher occurrence of postoperative complications, including anastomotic bleeding, incision infection, and anastomotic stenosis. The drainage of TDTs, to a certain extent, can support the anastomotic stoma and can be used to detect complications such as anastomotic bleeding and anastomotic infection early, which allows clinicians to take action timely. By using anoscopes and other instruments under direct vision, at the same time, TDTs with moderate hardness was selected, which can minimize the injury of the anastomosis. TDTs of appropriate size and hardness will not cause injury and bleeding of anastomosis. Hence, in cases of AL, the placement of an economical, efficient, and secure TDTs can be employed as a measure to mitigate the extent of AL.

Positive results were observed exclusively in subgroup analysis conducted on area, revealing that TDTs placement served as a protective factor for AL in the Asian group, whereas it posed a risk in the European group. This outcome could potentially be attributed to variations in the study's sample size, discrepancies in the assessment of AL, and the utilization of diverse types of TDTs.

Several factors contribute to AL, and more studies are being conducted to determine the causes and development of AL. It has been confirmed that some factors are closely related to AL's development, such as albumin levels lower than 4 g/dL [23] and operation time longer than 3 h [5]. As a common clinical treatment, the placement of TDTs has low technical requirements and is suitable for hospitals of every level. Multiple studies have documented the occurrence of unfavorable incidents associated with the placement of TDTs subsequent to RC surgery, with anal pain being the most frequently reported complication [4]. Due to the lack of comprehensive documentation regarding adverse events following TDTs placement in the original literature included in this meta-analysis, statistical analysis pertaining to such events was not performed in this study. The visual analogue scale was employed to assess the pain perception experienced by the patients, which is also suitable for the assessment of anal pain after the placement of TDTs. The score ranges from 0 to 10, where 0 denotes the absence of pain and 10 signifies the most severe pain that can be imagined [24]. The pain was subsequently categorized into four distinct levels. No pain: score of 0, indicating the absence of pain; Mild pain: score of 1–3, representing pain that is tolerable; Moderate pain: score of 4–6, indicating pain that may disrupt sleep but remains tolerable; Severe pain: score of 7–10, signifying pain that is unbearable. A randomized controlled study found that TDTs placement caused anal pain in 46.4% of patients, moderate pain in 3.9%, and unbearable pain in 3 patients [4]. What's more, other studies have documented iatrogenic perforation resulting from the placement of TDTs [6, 25], as well as cases necessitating emergency laparotomy due to such perforations [25]. In addition, studies have reported that no expected drainage effect occurs after the placement of TDTs, manifested as fecal discharge from the anus rather than from the TDTs [4].Despite the lack of correlation between the placement of TDTs and the occurrence of AL after RC surgery. However, it is considered that the placement of TDTs can reduce the severity of AL, and healthcare professionals can enhance patient outcomes by proactively optimizing preoperative nutrition, limiting surgical duration to a maximum of three hours, and implementing the placement of TDTs following AL. The placement of TDTs helps to discharge the intestinal contents in time, which is conducive to reducing the length of hospital stay. Studies have found that patients with high-risk factors for anastomotic leakage, such as diabetes and open surgery, have a higher probability of readmissions within 30 days [26]. The placement of TDTs positively affects the timely and rapid detection of intestinal abnormalities. Thus, the disease can be treated earlier and the reoperation rate can be reduced.

There are several strengths of this meta-analysis: All relevant prospective studies (*n* = 7) from recent years with rich data and high statistical power were included. In addition, our study included recently published randomized controlled trials and more participants (*n* = 1774) than previous meta-analyses. Finally, a sensitivity analysis was performed to assess the potential influence of utilizing adjusted risk ratios on the aggregated effect estimates.

There remain certain limitations within this study. Primarily, the present meta-analyses were unable to mitigate heterogeneity, whether in the overall population or in subgroup analyses. Furthermore, while gender and age are commonly recognized as confounding factors in numerous studies, there exist additional variables that may also hold significant importance, such as the type of TDTs, presence of diverting stoma, and utilization of Neoadjuvant therapy, which may also possess considerable significance. However, none of these phenomena have been thoroughly investigated. The third aspect pertains to the highly intricate and diverse nature of AL. The existing model is incapable of mitigating this heterogeneity. Fourth, the sample size of some included documents is small, and the statistical impact may exhibit constraints, thereby posing challenges in terms of generalizability of the findings.

## Conclusions

In conclusion, the placement of TDTs does not yield significant results in terms of reducing the occurrence of AL after RC surgery, including Grade B AL, and Grade C AL. Furthermore, TDTs placement does not lead to heightened complications such as anastomotic bleeding, incision infection, or anastomotic stenosis. Based on the potential for anal pain, iatrogenic perforation, and limited efficacy associated with TDTs placement, we advise against the immediate placement of TDTs following RC surgery. The findings of this research are derived from a compilation of seven prospective studies. Given the current scarcity of data and the variability observed among studies, the conclusion remains subject to scrutiny. Consequently, future investigations should prioritize the implementation of meticulously planned randomized controlled trials with substantial sample sizes to corroborate this assertion.

## Data Availability

The raw data supporting the conclusions of this article will be made available by the authors, without undue reservation.

## Abbreviations

TDTs: Transanal drainage tubes
AL: Anastomotic leakage
RC: Rectal cancer
RRs: Risk ratios
CI: Confidence intervals
